# Actin Beta-Like 2 as a New Mediator of Proliferation and Migration in Epithelial Ovarian Cancer

**DOI:** 10.3389/fonc.2021.713026

**Published:** 2021-09-23

**Authors:** Nicole Elisabeth Topalov, Doris Mayr, Clemens Scherer, Anca Chelariu-Raicu, Susanne Beyer, Anna Hester, Fabian Kraus, Mingjun Zheng, Till Kaltofen, Thomas Kolben, Alexander Burges, Sven Mahner, Fabian Trillsch, Udo Jeschke, Bastian Czogalla

**Affiliations:** ^1^ Department of Obstetrics and Gynecology, University Hospital, LMU Munich, Munich, Germany; ^2^ Institute of Pathology, Faculty of Medicine, LMU Munich, Munich, Germany; ^3^ Department of Medicine I, University Hospital, LMU Munich, Munich, Germany; ^4^ 5DZHK (German Centre for Cardiovascular Research), Partner Site Munich Heart Alliance, University Hospital, LMU Munich, Munich, Germany; ^5^ Department of Obstetrics and Gynecology, University Hospital Augsburg, Augsburg, Germany

**Keywords:** actin beta-like 2, nuclear factor of activated T-cells 5, epithelial ovarian cancer, prognosis, proliferation, migration

## Abstract

The impact of Actin beta-like 2 (ACTBL2), a novel described actin isoform, on epithelial ovarian cancer (EOC) biology has not been investigated so far. In this study, we analyzed the prognostic and functional significance of ACTBL2 and its regulatory element Nuclear factor of activated T-cells 5 (NFAT5). The expression of ACTBL2 and NFAT5 was examined in tissue microarrays of 156 ovarian cancer patients by immunohistochemistry. Aiming to assess the molecular impact of ACTBL2 on cellular characteristics, functional assays were executed *in vitro* upon siRNA knockdown of *ACTBL2* and *NFAT5*. ACTBL2 expression was identified as an independent negative prognostic factor for overall survival of EOC patients. EOC cell lines showed a significantly increased mRNA and protein level of ACTBL2 compared to the benign control. *In vitro* analyses upon siRNA knockdown of *ACTBL2* displayed a significantly reduced cellular viability, proliferation and migration. siRNA knockdown of *NFAT5* proved a significant molecular interplay by inducing a downregulation of ACTBL2 with a thus resulting concordant alteration in cellular functions, predominantly reflected in a decreased migratory potential of EOC cells. Our results provide significant evidence on the negative prognostic impact of ACTBL2 in EOC, suggesting its crucial importance in ovarian carcinogenesis by modulating cellular motility and proliferation.

## Introduction

Epithelial ovarian cancer (EOC) is the fifth leading lethal tumor entity in women and the most common cause of death among gynecological cancer patients (1). Due to comparably insufficient screening methods and minor clinical symptoms with a consecutively late diagnosis of advanced tumor stages, EOC is associated with a relatively low 5-year survival rate of less than 45% (2). Established and reliable prognostic factors for overall survival of EOC patients include the disease stage at diagnosis (FIGO), tumor grading, histological subtypes and patient’s age, with the volume of residual disease after primary surgery being the most significant one (3–6). First-line therapy consists of cytoreductive surgery and adjuvant platinum-based chemotherapy in the clinical course. This is followed by the use of bevacizumab or poly-ADP-ribose-polymerase inhibitors, as a recent promising therapeutic approach in the maintenance treatment of patients with at least partial response to chemotherapy (7, 8). While other gynecological tumor entities such as endometrial and cervical cancer are comparably prone to respond to immune therapy, no promising prognostic benefit in terms of ovarian cancer treatment has been shown yet (9–12). Despite new emerging therapeutic strategies in the past few years, widely accepted and reliable biomarkers for ovarian cancer are still rare due to lacking profound knowledge on molecular pathological mechanisms enhancing tumor development and progression.

Actin beta-like 2 (ACTBL2), a novel described actin isoform showing 92% structural similarity to ß-actin, was found to be a putative risk gene in ovarian cancer (13–15). Yet, the cellular function of ACTBL2 in EOC and its carcinogenetic impact on gynecological malignancies are thus far unknown. Despite the relatively high structural congruence to ß-actin, phylogenetic analyses revealed a genetic distance from other commonly known isoforms, with ACTBL2 being expressed in different cellular localizations and executing individual molecular functions (16, 17). A significant upregulation of ACTBL2 was yet detected in pancreatic ductal adenocarcinoma and colorectal cancer (18, 19). Moreover, a high abundance of ACTBL2 in hepatocellular carcinoma was associated with altered cellular growth properties and an impaired postoperative disease-free survival of affected patients (16). Mazur et al. identified ACTBL2 as a binding partner of gelsolin in melanoma cells, being part of cellular lamellipodia and thus hinting at its intracellular function and putatively promigratory effect (20). Additionally, functional assays revealed an impaired migration of vascular smooth muscle cells (VSMCs) after gene silencing of *ACTBL2* (21). In silico analyses focusing on the promotor sequence of *ACTBL2* displayed several putative binding sites for Nuclear factor of activated T-cells 5 (NFAT5) (21). Executing its manifold functions as a transcription factor, NFAT5 is required in regulating the expression of genes involved in controlling cellular osmotic stress and in orchestrating cellular migration and proliferation (22–25). Gene knockdown of *NFAT5* in vascular smooth muscle cells resulted in a significantly diminished ACTBL2 expression, proving their direct interaction (21). Apart from studies focusing on promigratory effects in biomechanically activated VSMCs, the regulatory impact of NFAT5 on ACTBL2 in tumor cells and the extent of the consequently provided alterations of cellular functions remain still unknown.

The present study aimed at elucidating the functional role of ACTBL2 and NFAT5 in epithelial ovarian cancer, intentionally assisting to obtain new findings on its etiology with regard to carcinogenetic and disease-promoting mechanisms.

## Material and Methods

### Ethical Approval

This study was approved by the Ethics Committee of the Ludwig-Maximilians-University (LMU), Munich, Germany (approval number 227-09, 18-392 and 19-972). All tissue samples used were obtained from material initially utilized for pathological diagnostics from the archives of the LMU, Munich, Germany. The diagnostic procedures were completed before the present study was performed, with the observers being fully blinded to the patients’ data during all experimental and statistical analyses. All experiments described were performed respecting the standards of the Declaration of Helsinki (1975).

### Patients and Specimens

Tissue microarrays of 156 EOC patients who underwent cytoreductive surgery between 1990 and 2002 at the Department of Obstetrics and Gynecology, Ludwig-Maximilians-University in Munich, Germany, were analyzed in the given study (**Table 1**). In previously performed studies regarding the present cohort, various other pathological parameters were investigated, thus enabling the execution of correlation analyses. The clinical data was obtained from the patients’ charts with the according follow-up data being received from the Munich Cancer Registry (MCR). Only patients with pathologically validated epithelial ovarian cancer were included, whereas benign as well as borderline tumors were accordingly excluded from the collective. Moreover, none of the considered patients had neoadjuvant chemotherapy in the clinical course. All samples used were formalin-fixed and paraffin-embedded (FFPE) before being examined by gynecological pathologists at the Department of Pathology, LMU, regarding clinical and pathological criteria. The samples were classified into histological subtypes [serous (n=110), clear cell (n=12), endometrioid (n=21), mucinous (n=13)] as well as rated by tumor grading, respecting the currently valid WHO classifications. Serous ovarian cancer was divided into low and high grading, while tissue samples of endometrioid histology were graded according to G1 to G3. For mucinous ovarian carcinoma, there is no explicit WHO classification; however, this subtype is often classified into G1 to G3 analogous to endometrioid subtype. Clear cell ovarian cancer was always categorized as G3. Further, staging was performed using the FIGO classification [I (n=35), II (n=10), III (n=103), IV (n=3)], while data on primary tumor extension according to the TNM classification was available in 155 cases showing the following distribution: T1 (n=40), T2 (n=18) and T3 (n=97). Concerning lymph node involvement, data was obtainable in 95 cases [N0 (n=43), N1 (n=52)], whereas data on distant metastasis was only accessible in 9 cases [M0 (n=3), M1 (n=6)]. Information on grading and FIGO stage is missing in 12 respectively 5 cases.

**Table 1:** Clinicopathological characteristics of ovarian cancer patients considered in this study.

Clinicopathological parameters	n	Percentage (%)
*Histology*		
serous	110	70.5
clear cell	12	7.7
endometrioid	21	13.5
mucinous	13	8.3
		
*Primary tumor expansion*		
TX	1	0.6
T1	40	25.6
T2	18	11.5
T3	97	62.3
		
*Nodal status*		
pNX	61	39.1
pN0	43	27.6
pN1	52	33.3
		
*Distant metastasis*		
pMX	147	94.2
pM0	3	1.9
pM1	6	3.8
		
*Grading serous*		
low	24	21.8
high	80	72.7
		
*Grading endometrioid*		
G1	6	28.6
G2	5	23.8
G3	8	38.1
		
*Grading mucinous*		
G1	6	46.2
G2	6	46.2
G3	0	0
		
*Grading clear cell*		
G3	12	100.0
		
*FIGO*		
I	35	22.4
II	10	6.4
III	103	66.0
IV	3	1.9
		
*Age*		
≤60 years	83	53.2
>60 years	73	46.8

### Immunohistochemistry

After dewaxing the formalin-fixed and paraffin-embedded ovarian cancer tissue microarrays in xylol for 20 minutes, the slides were shortly washed in 100% ethanol. Intending to avoid unspecific staining, the endogenous peroxidase was blocked by using 3% H_2_O_2_ in methanol for 20 minutes, before rehydrating the samples in descending concentrations of ethanol (100%, 70% and 50%) and shortly resting them in distilled water. Next, the slides were put in a pressure cooker filled with boiling sodium citrate buffer (pH=6) consisting of 0.1 M citric acid and 0.1 M sodium citrate and were consecutively heated for 5 minutes. Cooled down, the tissue samples were shortly washed in distilled water and then in phosphate-buffered saline (PBS) twice for 2 minutes each. To prevent an unspecific staining reaction during the course, the slides were incubated with a blocking solution [Reagent 1; ZytoChem Plus HRP Polymer System (mouse/rabbit), Zytomed, Berlin, Germany] for 5 minutes at room temperature (RT) followed by an overnight incubation of 16 hours at 4°C with the following primary antibodies: anti-ACTBL2, 1:800 dilution in PBS (rabbit IgG, polyclonal, abcam, ab100869), anti-NFAT5, 1:200 dilution in PBS (rabbit IgG, polyclonal, Sigma, HPA069711-100UL). Afterwards, the samples were again washed twice in PBS and subsequently treated with a post block reagent (Reagent 2; ZytoChem Plus HRP Polymer System (mouse/rabbit), Zytomed, Berlin, Germany) for 20 minutes at RT. After repeating the previously described washing step with PBS, the slides were incubated with an HRP-polymer containing bound anti-mouse as well as anti-rabbit antibodies (Reagent 3; ZytoChem Plus HRP Polymer System (mouse/rabbit), Zytomed, Berlin, Germany) for 30 minutes. For visualization, 3,3’diaminobenzidine (DAB) and the according substrate buffer (Liquid DAB and Substrate Chromogen System, DAKO, Munich, Germany) were applied on the tissue for 30 seconds (ACTBL2) and 1,5 minutes (NFAT5), respectively. The reaction was stopped by washing the slides in distilled water, followed by a counterstaining with Mayer’s acidic hemalum (Waldeck, Münster, Germany). After dehydrating the ovarian cancer tissue in a series of ethanol with ascending concentrations (70%, 96% and 100%), the slides were placed in xylol and finally covered. Kidney and vulva tissue served as negative and positive controls to examine the specificity of the immunoreaction as well as to assess the most suitable dilution of the used primary antibodies (**Figure S1**). Concerning the negative controls, the primary antibodies were each replaced by a specific isotype control antibody (BioGenex, Fremont, CA, USA).

### Immunocytochemistry

For immunocytochemistry (ICC) of ACTBL2 and NFAT5, assessing the basal protein expression in ovarian cancer cells, 1×10^6^ UWB1.289 cells were seeded on sterile microscope slides and maintained in culture as described below for 24 hours. After washing with PBS twice for 5 minutes each, the slides were fixed by being placed in 100% ethanol and methanol (1:1) at room temperature (RT) for 15 minutes and were subsequently air dried. Intending to avoid unspecific background staining, the slides were treated with a goat-derived serum (Vectastain Elite rabbit-IgG-kit, Vector Laboratories, Burlingame, CA, USA) for 20 minutes at RT after being rehydrated in PBS for 5 minutes. Next, the slides were incubated overnight for 16 hours at 4°C with the primary antibodies mentioned above in a 1:400 (ACTBL2) respectively 1:50 (NFAT5) dilution. Afterwards, the slides were washed in PBS for 5 minutes followed by a 30 minute incubation with a biotinylated secondary anti-rabbit antibody (Vectastain Elite rabbit-IgG-kit, Vector Laboratories, Burlingame, CA, USA) at RT. Again, the slides were washed in PBS and subsequently treated with an avidin-biotin-peroxidase complex (Vectastain Elite rabbit-IgG-kit, Vector Laboratories, Burlingame, CA, USA) for 30 minutes at RT. To finally visualize the staining, chromogen 3-amino-9-ethylcarbazole (AEC^+^, DAKO, Hamburg, Germany) was applied for 10 minutes at RT. In order to stop the reaction, the slides were placed in distilled water before being counterstained with Mayer’s acidic hemalum (Waldeck, Münster, Germany). After being washed in distilled water, the slides were covered using an aqueous mounting medium (Aquatex, Merck, Darmstadt, Germany).

For ICC of ACTBL2 and NFAT5 after gene silencing, 5×10^4^ UWB1.289 cells were seeded in each well of sterile 4-well chamber slides (Lab-Tek II Chamber Slides, Thermo Fisher Scientific, Denmark) and maintained in culture overnight. siRNA knockdown of *ACTBL2* respectively *NFAT5* was performed for 48 hours as explained below, before executing the immunocytochemical staining as previously described.

### Staining Evaluation and Statistical Analysis

The examination of all EOC specimens was performed using a Leitz photomicroscope (Wetzlar, Germany) with the immunohistochemical (IHC) staining being analyzed by applying the semi-quantitative immunoreactive score (IRS) (26). The score is calculated by multiplying the percentage of positively stained cells (0=no staining, 1 ≤ 10%, 2 = 11-50%, 3 = 51-80% and 4≥81%) by the predominating optical staining intensity (0=no, 1=weak, 2=moderate, 4=strong). For each staining performed, the immunoreactive score was obtained considering the distinct distribution pattern of the analyzed proteins. As separate scores were calculated for each cellular compartment, NFAT5 staining was assessed in the cytoplasm and the nucleus, whereas ACTBL2 expression was evaluated in the cytoplasm and the cell membrane.

For statistical analyses of all data obtained, IBM SPSS Statistics 26.0 (IBM Corporation, Armonk, NY, USA) was used. Spearman’s analysis (27) was performed to calculate bivariate correlations between the examined proteins and clinicopathological data. Further, Kruskal-Wallis-H test (28) was used to assess and compare the distribution of more than two independent samples in the analyzed collective. Overall survival of EOC patients was compared by executing log-rank testing with Kaplan-Meier curves being used for visualization (29). For identification of appropriate cut-off values in survival analyses, a ROC curve analysis was performed, being a reliable and widely accepted method for cut-off point selection (30). The Youden index, being defined as the maximum (sensitivity+specificity-1), was used to ensure the optimal cut-off, maximizing the sum of sensitivity and specificity (31, 32). For multivariate analyses, a Cox regression model of the investigated parameters was established (33). qPCR results were analyzed for statistical significance by using the obtained Ct values and calculating the relative expression by applying the 2^-ΔΔCt^ formula (34). Further *in vitro* experiments were statistically analyzed by performing Wilcoxon test with all *in vitro* analyses being visualized using GraphPad Prism 7.00 (San Diego, CA, USA). For all analyses, p-values ≤0.05 were considered to be statistically significant.

### Cell Lines

The human ovarian cancer cell lines ES-2 (clear cell), OVCAR3 (serous), TOV112D (endometrioid) and UWB1.289 (serous, BRCA1 negative) were obtained from ATCC (Rockville, MD, USA) and were maintained in culture using RPMI 1640 GlutaMAX Medium (Gibco, Paisley, UK) supplemented with 10% fetal bovine serum (FBS; Gibco, Paisley, UK) in a humified incubator at 37°C and 5% CO2. For reference, the benign human cell line HOSEpiC (ATCC, Rockville, MD, USA) was cultured in Ovarian Epithelial Cell Medium (OEpiCM; ScienCell, Carlsbad, CA, USA) according to the instructions of the company in a humified incubator at 37°C and 5% CO2. All cell lines used in this study were tested negative for mycoplasma in advance.

### qPCR

mRNA isolation was executed using the RNeasy Mini Kit (Qiagen, Venlo, Netherlands) according to the manufacturer’s protocol. Next, 1µg RNA was converted into cDNA using the MMLV Reverse Transcriptase 1st-strand cDNA Synthesis Kit (Epicentre, Madison, WI, USA). The mRNA expression of both ACTBL2 and NFAT5 was quantified by qPCR applying FastStart Essential DNA Probes Master and gene-specific primers (Roche, Basel, Switzerland, **Table S1**), with their relative expression being subsequently calculated by the 2-ΔΔCt formula using GAPDH as a housekeeping gene.

### siRNA Knockdown

UWB1.289 cells were transfected with small interfering RNA (siRNA) for *ACTBL2* and *NFAT5*, respectively (GeneSolution siRNA, Qiagen Sciences, MD, USA; for detailed information on the according sequences, see **Tables S2A, B**), by using Lipofectamine RNAiMAX reagent (Invitrogen, Carlsbad, CA, USA). For reference, a scrambled negative control siRNA (AllStars Negative Control siRNA, Qiagen, Hilden, Germany) was utilized. At first, 2,5×105 UWB1.289 cells/well were seeded on sterile 6-well plates and maintained in culture as described above. After reaching a cell density of 60-70%, the transfection was performed by treating the cells with OptiMEM Reduced Serum Medium (Thermo Fisher Scientific, Waltham, MA, USA) containing the siRNA-Lipofectamine complex. After 48 hours of incubation at 37°C and 5% CO2, the cells were harvested and used for further experiments. To prove the successful gene silencing, mRNA isolation and qPCR were subsequently executed as outlined above. Immunocytochemistry was applied as previously described to confirm the knockdown of ACTBL2 respectively NFAT5 on a protein level. Each siRNA knockdown was repeated and thus validated three times.

### Western Blot

For basal expression analysis of ACTBL2, untreated adherent UWB1.289 cells were lysed for 15 minutes at 4°C using 300µl RIPA buffer (Sigma Aldrich Co., St. Louis, MO, USA) containing a previously 1:100 diluted protease inhibitor (Sigma Aldrich Co., St. Louis, MO, USA). After adding 100µl of 4x Laemmli sample buffer, the protein samples were loaded and separated according to their molecular weight using a 10% sodium dodecyl sulphate-polyacrylamide gel (Mini-PROTEAN TGX Precast Gels, Bio-Rad Laboratories Inc., Hercules, CA, USA) at a voltage of 70 V for 2 hours. After transferring the proteins onto a polyvinylidene fluoride membrane (Sequi-Blot PVDF Membrane, Bio-Rad Laboratories Inc., Hercules, CA, USA) for 65 minutes at 145mV and 4°C, the membrane was blocked for 1 hour at RT in 5% milk powder solution to prevent an unspecific antibody reaction. Subsequently, the membrane was incubated gently shaking overnight at RT with the following diluted primary antibodies: anti-ACTBL2 (1:500 dilution; rabbit IgG, polyclonal, abcam, ab100869) and ß-actin (1:1000 dilution; mouse IgG, monoclonal, Sigma, St. Louis, USA) with ß-actin serving as a control. Afterwards, the membranes were washed three times with TBS/Tween and subjected to the corresponding species-specific secondary antibodies (goat-anti-rabbit/mouse, 1:1000 dilution, Jackson Immuno Research, UK) for 1 hour at RT. After repeating the previously described washing steps, the antibody complexes were visualized using 5-bromo-4-chloro-3-indolylephosphate/nitro-blue-tetrazolium chloride (BCIP/NBT, Promega) in 0.1M Tris-HCl and 0.15M NaCl for 5-10 minutes. Western blotting analysis was performed using the Bio-Rad Universal Hood II and the corresponding software (Quantity One; Bio-Rad Laboratories Inc., Hercules, CA, USA). Each Western blot was repeated three times.

### Cell Viability Assay and Proliferation Assay

For cell viability measurements a 3-(4,5-dimethylthiazol-2-yl)-2,5-diphenyltetrazolium bromide (MTT, Sigma, M-5655, 500 mg) colorimetric assay was conducted, while changes in cell proliferation were detected by performing a 5-bromo-2-deoxyuridine (BrdU) assay (Roche Cell Proliferation Elisa, BRDU (Colorimetric), Roche, Basel, Switzerland). In each assay executed, 5×103 UWB1.289 cells/100µl were seeded on sterile 96-well plates and maintained in culture overnight using RPMI 1640 GlutaMAX medium with 10% FBS. Subsequently, gene silencing of ACTBL2 respectively NFAT5 was performed as previously described. After 72h, both MTT and BrdU assay were conducted according to the manufacturer’s protocol. An Elx800 universal Microplate Reader (BioTek, Winooski, VT, USA) was used to measure the optical density (OD) in each well at 595nm (MTT) and 450nm (BrdU). Each experiment was repeated and thus validated three times.

### Wound Healing Assay

To analyze the cellular migration after gene silencing, 8×10^5^ UWB1.289 cells/well were seeded on sterile 6-well plates and maintained in culture as previously outlined. After 24h, a sterile 200µl pipet tip was used to scratch a vertical line centrally into the monolayer, aiming to create an artificial wound. After gently washing the cells with PBS to remove excess cells, siRNA knockdown of each *ACTBL2* and *NFAT5* was performed as described above. To consequently monitor the cellular migration, digital images of each scratch were taken exactly 0h, 24h and 48h after the transfection by using an inverse phase-contrast microscope (Leica Dmi1, Leica, Wetzlar, Germany) and the according camera (Leica MC120 HD, Leica, Wetzlar, Germany). The images were subsequently analyzed by measuring the wounded areas at each time using ImageJ (https://imagej.nih.gov/ij/). The relative cell migration was outlined by calculating the difference of the covered area at 24h and 0h as well as 48h and 0h, and comparing the results to the untreated control.

## Results

### ACTBL2 Expression in Epithelial Ovarian Cancer Correlates With Clinical and Pathological Characteristics

To examine the role of ACTBL2 in epithelial ovarian cancer, ACTBL2 expression was investigated in 156 specimens. Immunohistochemical staining of ACTBL2 was assessed in 134 cases (86%) in the cytoplasm and the cell membrane with a median (range) IRS of 4 (0,12) and 2 (0,8), respectively (**Tables S3A, B**). Positive ACTBL2 expression was defined and further investigated as combined cytoplasmic (IRS>2; n=117) and membranous (IRS>0; n=110) expression in the present cohort *via* ROC-curve analyses.

Consecutively performed correlation analyses of combined ACTBL2 expression and clinicopathological data revealed a significant positive correlation between high ACTBL2 expression and serous histology (**Table 2**; *p*=0.013, *Cc*=0.213). Moreover, high levels of ACTBL2 correlated significantly with high grading of serous carcinoma (**Table 2**; *p*=0.003, *Cc*=0.253).

**Table 2:** Correlation analysis of ACTBL2 expression and clinicopathological data.

Variables	Combined ACTBL2 expression	
	p	Correlation coefficient
Histology		
*serous*	0.013*	0.213
*clear cell*	0.044*	-0.174
*endometrioid*	0.176	-0.118
*mucinous*	0.640	-0.041
FIGO	0.728	0.031
pT	0.150	0.126
pN	0.883	0.016
Grading		
*serous – low grading*	0.098	-0.144
*serous – high grading*	0.003*	0.253
*clear cell, endometrioid and mucinous – G1 to G3*	0.589	0.096

Spearman’s correlation analysis of combined cytoplasmic (IRS>2) and membranous (IRS>0) ACTBL2 expression and clinicopathological characteristics, showing a positive correlation between positive ACTBL2 expression, serous histology (p=0.013, Cc=0.213) and high grading of serous carcinoma (p=0.003, Cc=0.253), respectively. Significant correlations are indicated with asterisks (*p < 0.05).

(p=two-tailed significance, Cc=correlation coefficient).

### Positive ACTBL2 Expression Is Associated With Impaired Overall Survival of EOC Patients

Intending to further investigate the prognostic significance of ACTBL2 expression in ovarian cancer, a univariate analysis of overall survival (OS) was performed.

In the present cohort, the patients’ median age was 58.7 (± 31.4) years with a range of 31-88 years, while their median OS was 34.4 (± 57.8) months.

**Figure 1:**
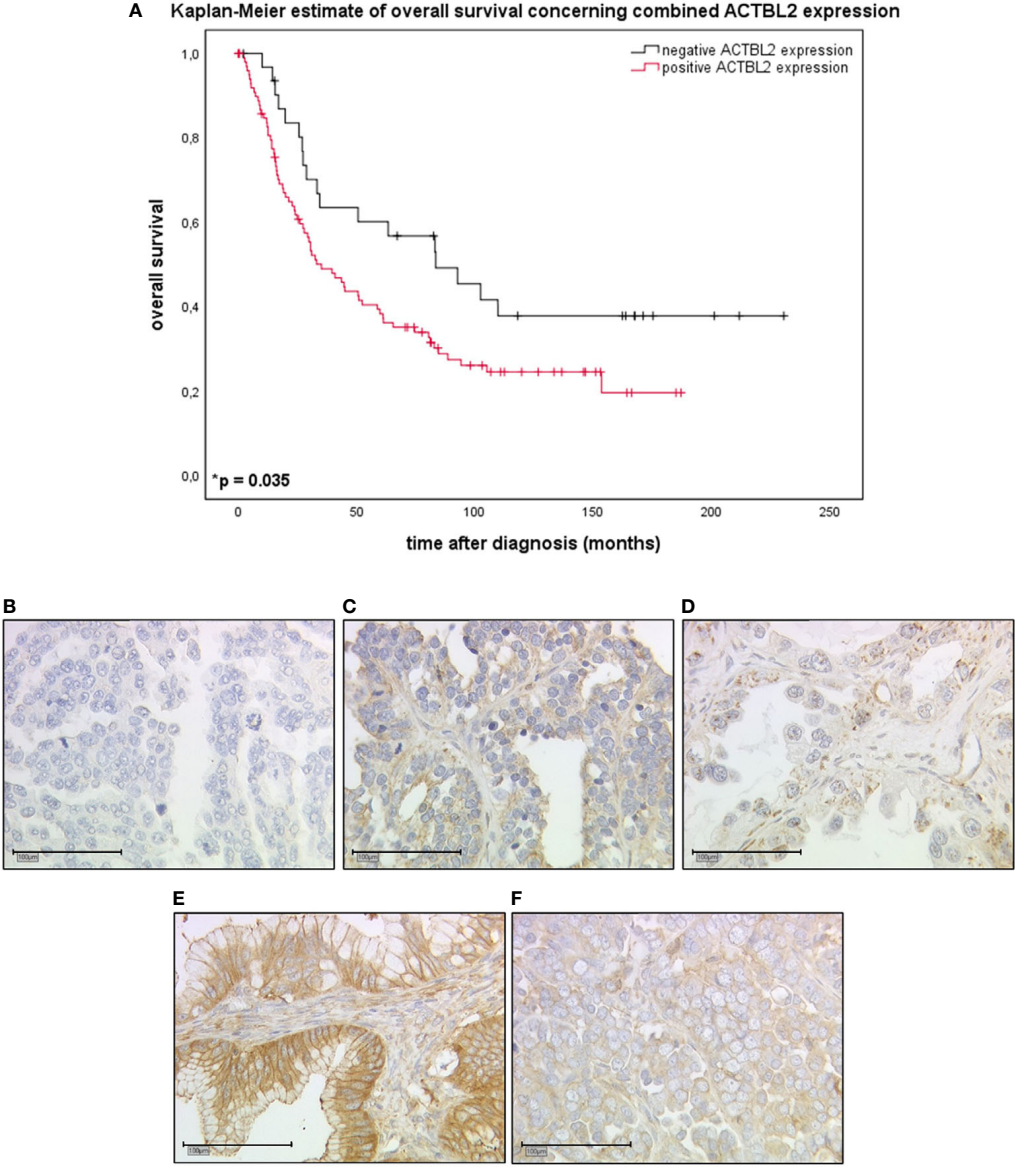
Kaplan-Meier estimate of combined ACTBL2 expression in EOC patients as detected by immunohistochemistry. **(A)** Kaplan-Meier estimate (log-rank testing) concerning combined cytoplasmic (IRS>2) and membranous (IRS>0) ACTBL2 expression in epithelial ovarian cancer, being associated with impaired overall survival (median OS 35.2 *vs*. 83.4 months; *p*=0.035). Censoring events were marked in the graphs (+). **(B–F)** Detection of ACTBL2 by immunohistochemistry. Exemplary photographs (25x magnification; scale bar=100µm), showing the differences between ACTBL2-negative **(B)** and ACTBL2-positive tissue of all histological subtypes of EOC **(C–F)**, thus visually supporting the survival analysis displayed above: **(B)** serous carcinoma, cytoplasmic IRS=0, membranous IRS=0; **(C)** serous carcinoma, cytoplasmic IRS=4, membranous IRS=4; **(D)** clear cell carcinoma, cytoplasmic IRS=4, membranous IRS=1; **(E)** mucinous carcinoma, cytoplasmic IRS=8, membranous IRS=8; **(F)** endometrioid carcinoma, cytoplasmic IRS=8, membranous IRS=4.

Combined cytoplasmic and membranous, thus positive ACTBL2 expression in EOC patients (n=101) was associated with a significantly shorter overall survival compared to patients with negative ACTBL2 expression (n=32; median OS 35.2 *vs*. 83.4 months; *p*=0.035) (**Figures 1A–F**).

### Positive ACTBL2 Expression and Clinicopathological Parameters Are Independent Prognostic Factors for Overall Survival

Aiming to detect which parameters are independent factors for overall survival in the present cohort, a multivariate Cox regression analysis was performed (**Table 3**). Patients’ age (*≤60 vs. >60* years; *p*=0.011) as well as FIGO stage (FIGO I, II *vs*. III, IV; *p*<0.001) were confirmed as independent prognostic factors. Additionally, positive ACTBL2 expression (*p*=0.013), as previously defined, was found to be a novel and statistically independent prognostic factor for impaired overall survival of ovarian cancer patients. In contrast, tumor histology and nodal status were not independent in the established model.

**Table 3:** Multivariate analysis.

Covariate	Hazard Ratio	95% CI	p-value
Patients’ age (*≤60 vs. >60*)	1.830	1.151-2.910	0.011*
Histology	0.980	0.726-1.321	0.892
FIGO (*I, II vs. III, IV*)	4.295	2.004-9.206	<0.001**
Nodal status (*pNX/0 vs. pN1*)	0,935	0.578-1.514	0.785
positive ACTBL2 expression	2.034	1.161-3.564	0.013*

Multivariate Cox regression analysis of ovarian cancer patients (n=156) and their clinicopathological characteristics considered in this study. Significant independent factors for overall survival in the present cohort are indicated with asterisks (*p < 0.05; **p < 0.001).

### *ACTBL2* Expression Is Significantly Elevated in Ovarian Cancer Cell Lines, Showing Highest Level in Serous UWB1.289 Cells

**Figure 2:**
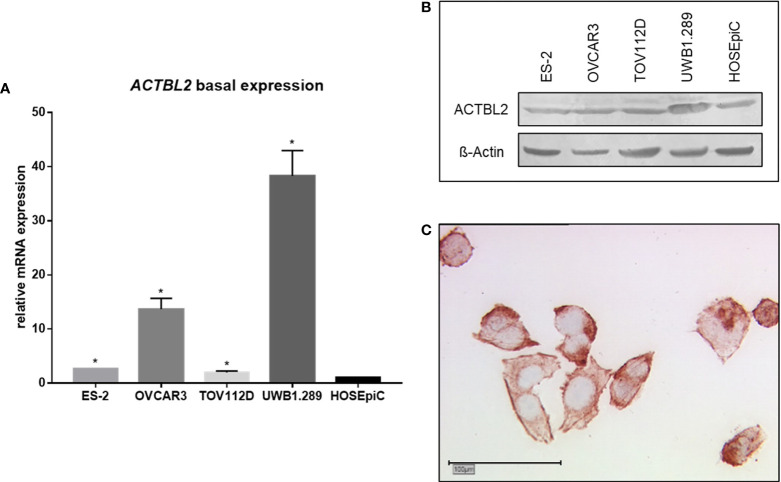
Basal expression of ACTBL2 in ovarian cancer cell lines. **(A)** qPCR results showing *ACTBL2* expression in four EOC cell lines (ES-2, OVCAR3, TOV112D, UWB1.289) compared to the benign control cell line (HOSEpiC; n=6; *p=0.028). **(B)** Exemplary Western blot analysis of ACTBL2 expression (42 kD) in four EOC cell lines (ES-2, OVCAR3, TOV112D, UWB1.289) compared to the benign control cell line (HOSEpiC). **(C)** Detection of ACTBL2 in UWB1.289 cells by immunocytochemistry. Exemplary photographs (25x magnification; scale bar=100µm) showing protein expression in both cytoplasm and cell membrane.

The basal mRNA expression of *ACTBL2* was analyzed by qPCR in four EOC cell lines as well as in the benign cell line HOSEpiC (**Figure 2A**). All malignant cell lines displayed a significantly elevated *ACTBL2* expression compared to the benign control (*p*=0.028). Supporting our aforementioned results in immunohistochemistry, both serous cell lines OVCAR3 and UWB1.289 showed higher levels of *ACTBL2* than tested tumor cells of other histological subtypes. The BRCA1 mutant cell line UWB1.289 showed the comparatively highest ACTBL2 expression on mRNA as well as on protein level, whereas OVCAR3 cells showed a protein expression of ACTBL2 comparable to the non-serous cell lines used in this study (**Figure 2B**). Additionally executed immunocytochemical staining of UWB1.289 cells confirmed the cytoplasm and the cell membrane as locations of ACTBL2 expression, corroborating our findings from previous immunohistochemical analyses (**Figure 2C**).

### Downregulation of *ACTBL2 In Vitro* Decreases Viability, Proliferation and Migration of UWB1.289 Cells, Indicating Its Functional Role in Serous Ovarian Cancer

Intending to elucidate the cellular function of ACTBL2 in terms of ovarian cancer etiology and progression, further *in vitro* experiments were performed. Since UWB1.289 cells showed the highest level of *ACTBL2*, this cell line was selected for additional investigations upon targeted gene silencing.

After proving the successful downregulation of ACTBL2 by both qPCR and immunocytochemistry (**Figure S2**), functional assays were executed to assess its impact on tumor cell biology. Given our previously described findings, we hypothesized that ACTBL2 might enhance cellular viability, proliferation and migration in ovarian cancer, thus serving as a potential explanation for the poor prognosis associated with positive ACTBL2 expression in EOC patients.

**Figure 3:**
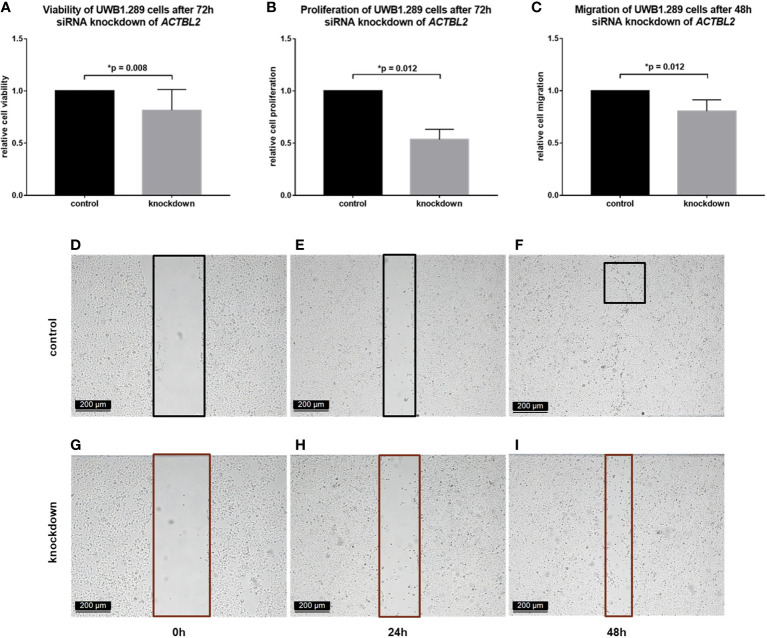
Functional assays after *ACTBL2* silencing in UWB1.289 cells. **(A)** MTT assay results after siRNA (sequence 3) knockdown of *ACTBL2*, showing a significantly reduced cell viability (n=9, *p*=0.008). **(B)** BrdU assay results, displaying a significantly decreased cell proliferation after silencing of *ACTBL2* (n=8, *p*=0.012) **(C–I)** Wound healing assay results **(C)** after siRNA knockdown of *ACTBL2*, showing a significantly reduced migration of UWB1.289 cells after 48h **(G–I)** compared to the untreated control (**D–F**; n=8, *p*=0.012) (10x magnification; scale bar=200µm).

In each assay performed, the results obtained in UWB1.289 cells after siRNA knockdown of *ACTBL2* were compared to the results of an untreated control. As shown in **Figure 3**, successful downregulation of ACTBL2 led to a significant decrease in cellular viability (**Figure 3A**; *p*=0.008). Moreover, *ACTBL2* silencing significantly inhibited the proliferation of ovarian cancer cells (**Figure 3B**; *p*=0.012). In addition, siRNA transfected cells displayed a significantly reduced migration as compared to the untreated group (**Figures 3C–I**; *p*=0.012).

Summarizing, our findings suggest that the downregulation of ACTBL2 results in a significant decrease in viability, proliferation and migration of ovarian cancer cells, inversely supporting our hypothesis regarding the cellular function of ACTBL2.

### Downregulation of *NFAT5 In Vitro* Regulates *ACTBL2* Expression and Consecutively Reduces Viability, Proliferation and Migration of UWB1.289 Cells

Aiming to assess molecular biological mechanisms regulating the function of ACTBL2, the impact of NFAT5 on ovarian cancer cells was further investigated.

**Figure 4:**
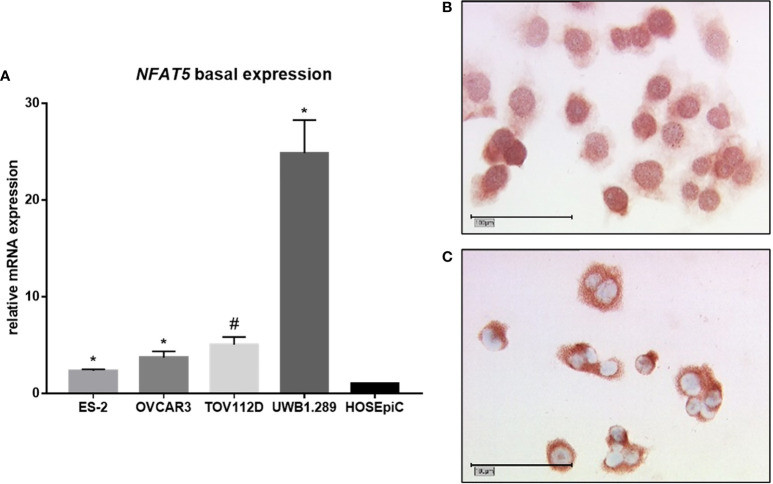
Basal expression of NFAT5 in ovarian cancer cell lines. **(A)** qPCR results showing *NFAT5* expression in four EOC cell lines (ES-2, OVCAR3, TOV112D, UWB1.289) compared to the benign control cell line (HOSEpiC; n=6; *p=0.028; #*p*=0.027). **(B, C)** Detection of NFAT5 in UWB1.289 cells by immunocytochemistry. Exemplary photographs (25x magnification; scale bar=100µm) showing protein expression in both nucleus **(B)** and cytoplasm **(C)**.

Firstly, the basal mRNA expression of *NFAT5* was analyzed by qPCR accordingly to our aforementioned experiment regarding the basal expression of *ACTBL2* (**Figure 4A**). Again, all malignant cell lines used in our study showed a significantly elevated *NFAT5* expression compared to the benign control cell line HOSEpiC (*p=0.028, *#p*=0.027). Reflecting our previously revealed results concerning the mRNA expression of *ACTBL2*, UWB1.289 cells showed the highest level of *NFAT5*. Supporting the assumption of NFAT5 functioning as a transcription factor, immunocytochemical staining of UWB1.289 cells confirmed both cytoplasm and nucleus as locations of NFAT5 expression (**Figures 4B, C**).

In addition, *in vitro* experiments in UWB1.289 cells were performed to characterize the functional connection between ACTBL2 and its putative regulatory element NFAT5.

**Figure 5:**
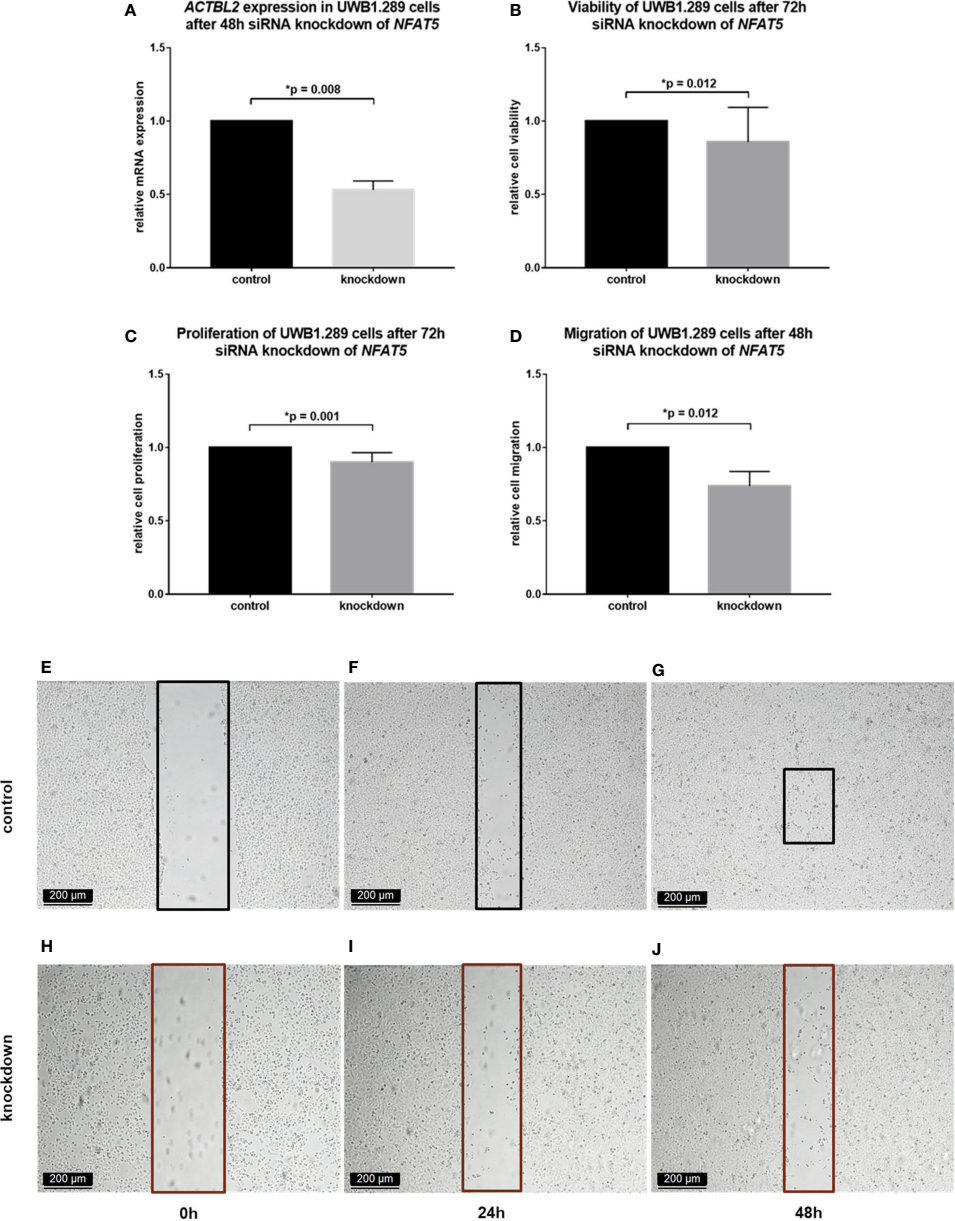
Functional assays after *NFAT5* silencing in UWB1.289 cells. **(A)** *ACTBL2* expression in UWB1.290 cells after 48h siRNA (sequence 7) knockdown of *NFAT5*, proving its successful downregulation on mRNA level compared to the untreated control (*p*=0.008). **(B)** MTT assay results after siRNA knockdown of *NFAT5*, showing a significantly reduced cell viability (n=8, *p*=0.012). **(C)** BrdU assay results, displaying a significantly decreased cell proliferation after silencing of *NFAT5* (n=15, *p*=0.001) **(D–J)** Wound healing assay results **(D)** after siRNA knockdown of *NFAT5*, showing a significantly reduced migration of UWB1.289 cells after 48h **(H–J)** compared to the untreated control (**E–G**; n=8, *p*=0.012) (10x magnification; scale bar=200µm).

In a first step, *NFAT5* silencing was induced in the selected cell line by siRNA transfection. The successful downregulation of NFAT5 on mRNA and protein level was proved by qPCR and immunocytochemistry, respectively (**Figure S3**). Moreover, the expression of *ACTBL2* after effectively performed *NFAT5* silencing was investigated by qPCR, showing a significant decrease of 46% after 48 hours (**Figure 5A**; *p*=0.008). Thus, we presumed that the downregulation of *ACTBL2* caused by *NFAT5* silencing would further lead to a decrease in cellular viability, proliferation and migration, reflecting our previously outlined results after *ACTBL2* knockdown. Consequently, we again performed the functional assays mentioned above, comparing the results obtained after siRNA knockdown of *NFAT5* to an untreated control. As shown in **Figure 5**, successful *NFAT5* silencing caused a significant decrease in cellular viability (**Figure 5B**; *p*=0.012) as well as significantly reduced cell proliferation rates (**Figure 5C**; *p*=0.001). Further, the downregulation of NFAT5, and consecutively *ACTBL2*, significantly inhibited the migration of UWB1.289 cells compared to the untreated control (**Figures 5D–J**; *p*=0.012).

In sum, our results show for the first time a functional relation between NFAT5 and ACTBL2 in ovarian cancer, with *NFAT5* silencing regulating the effect of ACTBL2 on cellular functions, predominantly resulting in a decreased migratory potential of UWB1.289 cells.

### Cytoplasmic NFAT5 Expression in Epithelial Ovarian Cancer Correlates With Prognostically Favorable Clinical and Pathological Characteristics

In order to evaluate its impact in a clinical relation, NFAT5 expression was investigated in the previously described patient cohort (n=156, **Table 1**). NFAT5 staining was assessed in 127 cases (81%) in the cytoplasm (**Figure S4**) with a median (range) IRS of 0 (0,8) (**Table S3C**), while nuclear expression was only detected in 2 cases. Hence, considering its function as a transcription factor, NFAT5 was mainly present in its inactive form in the analyzed collective.

**Figure 6:**
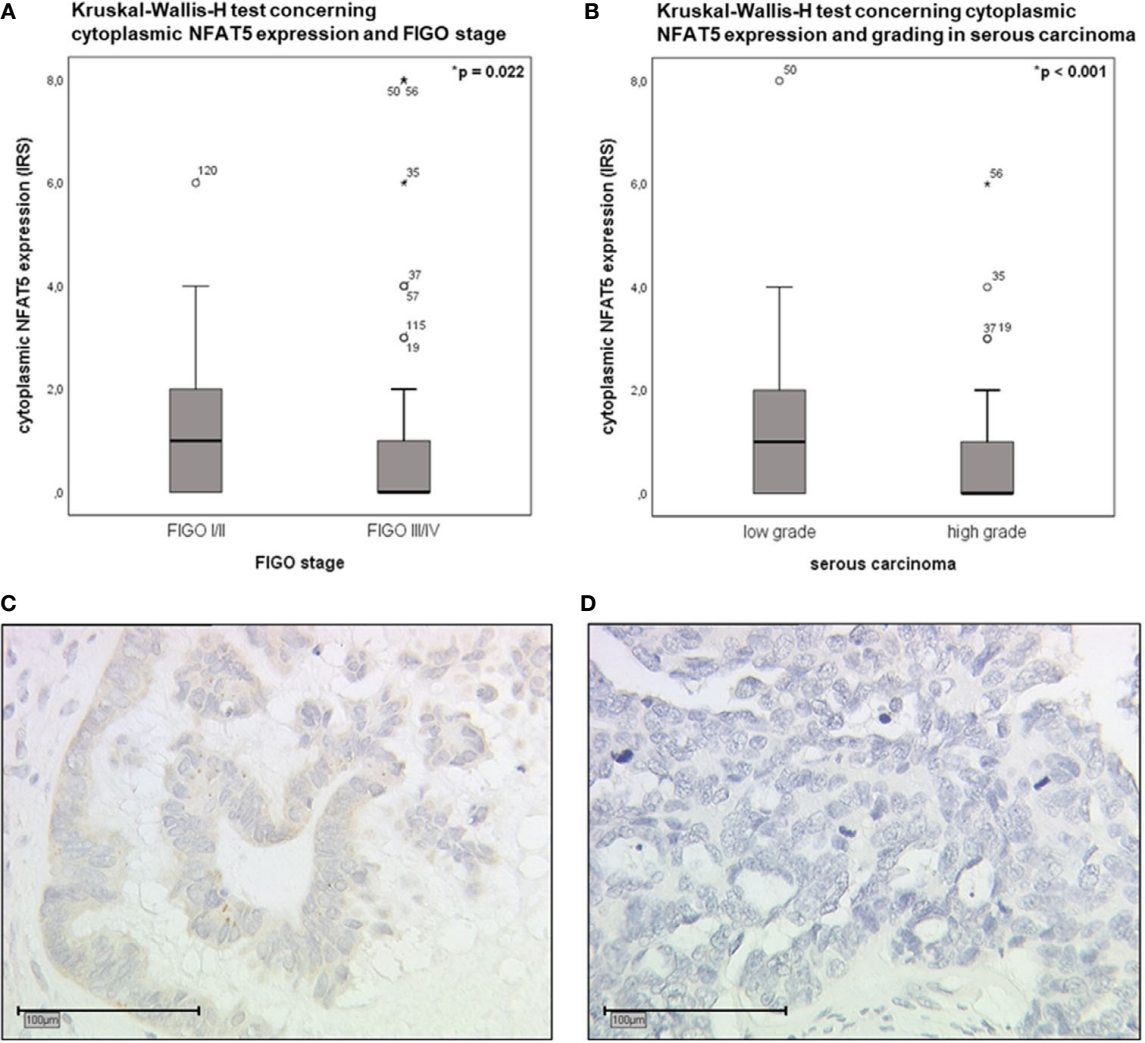
Boxplot analysis of cytoplasmic NFAT5 expression (Kruskal-Wallis-H test). **(A)** Boxplot analysis of cytoplasmic NFAT5 expression comparing FIGO I,II (n=37, median IRS=1) to FIGO III,IV (n=89, median IRS=0; *p*=0.022). **(B)** Boxplot analysis of cytoplasmic NFAT5 expression in serous carcinoma (n=89) comparing low grading (n=26, median IRS=1) to high grading (n=63, median IRS=0; *p*<0.001). **(C, D)** Exemplary photographs (25x magnification; scale bar=100µm) of cytoplasmic NFAT5 expression in serous histological subtype, comparing positive expression in low grade (**C**, IRS=4) to negative expression in high grade (**D**, IRS=0) carcinoma.

Additionally performed correlation analyses revealed significant correlations between cytoplasmic NFAT5 expression and clinicopathological characteristics (**Table S4**). Based on the thus detected results, Kruskal-Wallis-H tests were executed to further elucidate potential differences within FIGO stages and grading of serous carcinoma (**Figure 6**). Accordingly, low FIGO stages displayed a significantly higher cytoplasmic NFAT5 expression than advanced FIGO stages (**Figure 6A**; *p*=0.022). In addition, elevated cytoplasmic NFAT5 expression was significantly associated with low grading of serous carcinoma (**Figures 6B–D**; *p*<0.001).

Concluding, the presence of NFAT5 in the cytoplasm as an inactive transcription factor was linked to prognostically favorable clinical and pathological characteristics of epithelial ovarian cancer.

### High Gene Expression of *ACTBL2* and *NFAT5* in Large Independent EOC Cohorts Is Significantly Associated With Impaired Overall Survival

Aiming to validate the prognostic impact of *ACTBL2* and *NFAT5* on overall survival respecting a larger collective of EOC patients, the Kaplan-Meier Plotter database was used (35). For both genes respectively, patients were divided into high- and low-expression groups based on gene-specific cut-off values, before accordingly executing analyses concerning overall survival. The survival time of patients with high *ACTBL2* gene expression (n_high_=272) was shown to be significantly shorter compared to patients of the low-expression group (n_low_=101, *p*=0.036; **Figure S5A**), supporting our previously outlined results from immunohistochemical analyses regarding the prognostic relevance of cytoplasmic and membranous ACTBL2 expression in EOC patients.

Regarding *NFAT5* gene expression, comparable results were achieved by showing that high *NFAT5* gene expression (n_high_=152) is significantly correlated with an impaired prognosis of ovarian cancer patients (n_low_= 221; *p*=0.027; **Figure S5B**). Since the protein expression of NFAT5 was mainly detected in the cytoplasm of patients in our collective, being linked to prognostically favorable clinicopathological characteristics, a negative prognostic impact of nuclear NFAT5 expression can be assumed. Concordantly, although detected in very few cases in our cohort, nuclear protein expression of NFAT5 as a transcription factor of *ACTBL2* was associated with a significantly shorter overall survival of EOC patients (*p*=0.036; **Figure S5C**). However, as the survival analysis shown in **Figure S5B** is solely considering the gene expression of *NFAT5*, a more comprehensive analysis on the according protein distribution in each cellular compartment is required, to precisely allow a statement on its definite prognostic impact. An additionally performed correlation analysis of *ACTBL2* and *NFAT5* expression using the TIMER database (36) revealed a positive correlation trend between both genes (*Cc*=0.103, *p*=0.073; **Figure S5D**), hinting at their previously outlined functional relation in epithelial ovarian cancer.

## Discussion

In recent years, only very few studies have focused on Actin beta-like 2 and its molecular function. Whereas studies revealed an upregulation of ACTBL2 in pancreatic, colorectal and hepatocellular carcinoma, investigations focusing on its carcinogenetic impact in gynecological malignancies are still missing (16, 18, 19). Altered growth properties of hepatocellular carcinoma cells and a consecutively impaired disease-free survival of affected patients suggest a prognostic impact upon high intracellular protein abundance (16). By analyzing the expression pattern of Actin beta-like 2 in 156 EOC patients, we could show that ovarian cancer of high-grade serous histology displayed a significantly higher combined cytoplasmic and membranous ACTBL2 expression than specimens of other histological subtypes. Consistently, the combined and thus positive ACTBL2 expression was associated with an impaired overall survival of affected patients and additionally being confirmed as a novel independent prognostic factor. In summary, our study provides for the first time significant evidence on the prognostic relevance of ACTBL2 expression in epithelial ovarian cancer.

Aiming at further elucidating the molecular function of Actin beta-like 2 regarding disease-promoting hence survival-limiting mechanisms, we focused on comprehensive *in vitro* analyses. Experiments assessing the basal expression of *ACTBL2* revealed significantly elevated *ACTBL2* levels in all tested ovarian cancer cell lines compared to the benign control. Consistent with our shown results regarding the expression in EOC patients, serous UWB1.289 cells showed the comparably highest ACTBL2 abundance on mRNA and protein level each. Providing knowledge on its molecular function, targeted gene silencing of *ACTBL2* in the selected cell line resulted in a reduced protein expression and a consecutively decreased cellular viability and migration.

Mazur et al. identified Actin beta-like 2 as a binding partner of gelsolin in human melanoma cells (20). Gelsolin, a multifunctional actin-binding protein, was shown to be present in the edge of lamellipodia and thus structures enriched in filamentous actin and involved in cellular migration (20, 37). A high expression of gelsolin in colorectal carcinoma was shown to increase the cellular migratory potential (38). As the proximity between gelsolin and polymerization competent ACTBL2 in lamellipodia was shown to be close enough for direct interaction, a congruent promigratory effect of Actin beta-like 2 was hypothesized (20, 21). As previously outlined, ACTBL2 expression was detected in the membrane of EOC cells by IHC as well as immunocytochemically *in vitro* in UWB1.289 cells. Since only combined cytoplasmic and membranous expression had a significant impact on patients’ overall survival, the impaired prognosis might be based on the promigratory effect of Actin beta-like 2 provided by lamellipodia, being in line with pre-existing studies and supporting our results upon gene silencing of *ACTBL2*. Emphasizing the impaired OS of EOC patients upon positive ACTBL2 expression, a crucial favorable effect on metastatic processes can be assumed. Nonetheless, as the analyzed cohort contained very few cases of distant metastasis, more patients’ data is yet to be collected to further assess the contribution of ACTBL2 to enhanced cellular motility in the course of ovarian cancer development with special regard to metastatic mechanisms.

Further, we observed a decline of 46% in cellular proliferation upon *ACTBL2* gene knockdown. While recent studies have only focused on ACTBL2 in a promigratory context (16, 21), our findings suggest an additional enhanced proliferative effect of Actin beta-like 2 in ovarian cancer cells, simultaneously underlining the observed correlation between high ACTBL2 expression in EOC patients and comparably fast proliferating serous carcinoma of high-grade histology. Intending to reveal putatively counteracting mechanisms on the function of ACTBL2, we assessed the regulatory impact of NFAT5 based on studies executed in vascular smooth muscle cells (21). Hödebeck et al. showed that an siRNA induced gene knockdown of *NFAT5* resulted in a reduced cytoplasmic ACTBL2 expression of stretch stimulated VSMCs (21). NFAT5 itself is commonly known to be involved in enhancing cell migration and proliferation as well as to react to conditions of severe cellular osmotic stress (22–25). Nonetheless, the present study focused primarily on effects provided by NFAT5 upon ACTBL2 regulation. *In vitro* analyses of *NFAT5* in ovarian cancer revealed a significantly elevated mRNA expression in UWB1.289 cells, again being highest compared to other tested malignant cell lines. Protein expression of NFAT5 was detected in both nucleus and cytoplasm, reflecting its previously described function as a transcription factor of *ACTBL2* (21). Accordingly, downregulation of *ACTBL2* on mRNA level was successfully achieved by gene silencing of *NFAT5*. As viability and proliferation of UWB1,289 cells were consecutively diminished, a functional relation between NFAT5 and ACTBL2 in ovarian cancer was revealed for the first time. Moreover, a crucial role of ACTBL2 in cellular motility was again confirmed, reflected by a significantly declined cellular migration of 24% upon targeted *NFAT5* silencing. Taking clinical aspects into account, the presence of NFAT5 as an inactive transcription factor in EOC patients was linked to prognostically favorable characteristics, as a high cytoplasmic protein abundance correlated significantly with low FIGO stages and low grading of serous carcinoma.

Several studies provided evidence that nuclear translocation and activity of NFAT5 depend on posttranslational palmitoylation processes and are thus linked to cellular fatty acid oxidation (FAO) (39, 40). Targeted and irreversible inhibition of mitochondrial carnitine palmitoyltransferase 1 (CPT1) by Etomoxir resulted in a consecutively reduced cytoplasmic ACTBL2 abundance (21), since palmitoylation of NFAT5 was required to assure a nuclear entry within stretch-stimulated vascular smooth muscle cells (40). Apart from studies focusing on VSMCs, there is yet no evidence on the regulatory impact of Etomoxir on NFAT5 and ACTBL2 in cancer cells. The influence of FAO on carcinogenetic processes and consequently altered cellular functions upon irreversible CPT1 inhibition has been recently investigated in several tumor entities, demonstrating that Etomoxir might display a highly interesting and considerable therapeutic concept due to its antiproliferative effect (41–44). Nonetheless, Etomoxir was shown to simultaneously induce severe cellular oxidative stress *in vitro* (45) and *in vivo*, since a double-blind randomized phase II clinical trial on its therapeutic effect on congestive heart failure was prematurely stopped due to newly occurred hepatotoxicity (46). As our results confirmed a significant decrease in proliferation and migration of ovarian cancer cells upon specific downregulation of NFAT5 and ACTBL2, the irreversible blockade of CPT1 provided by Etomoxir might display a new and more precise antiproliferative approach in oncology, assumptively diminishing the therapy-limiting cytotoxicity upon systemic treatment. Since ACTBL2 expression was shown to be associated with an impaired prognosis of ovarian cancer patients, putatively enhanced by its promigratory characteristics, a reduction of intracellular levels of ACTBL2 might result in prognostically favorable alterations in tumor biology. However, further experiments are required to assess the potential of Etomoxir of being a new putative mechanism to directly counteract the effects of increased ACTBL2 expression in ovarian cancer cells.

Concluding, the present study investigated the carcinogenetic and prognostic impact of ACTBL2 and NFAT5 in epithelial ovarian cancer by elucidating their expression pattern in EOC patients and their functional molecular interplay *in vitro*. Our results suggest ACTBL2 and its regulatory element NFAT5 to be of significant functional and prognostic importance in ovarian carcinogenesis by modulating cellular proliferation and motility. Further studies evaluating the targeted antiproliferative use of Etomoxir are necessary to precisely analyze its impact on NFAT5 and ACTBL2 expression *in vitro* and *in vivo* with special regard to consecutively altered cellular functions in epithelial ovarian cancer.

## Data Availability Statement

The original contributions presented in the study are included in the article/**Supplementary Material**. Further inquiries can be directed to the corresponding author.

## Ethics Statement

The studies involving human participants were reviewed and approved by Ethics Committee of the Ludwig-Maximilians-University (LMU), Munich, Germany. The patients/participants provided their written informed consent to participate in this study.

## Author Contributions

Conceptualization, UJ, BC, and NT. Validation, DM, CS, AB, SM, and FT. Formal analysis, NT, UJ, and BC. Investigation, NT, UJ, and BC. Writing - original draft preparation, NT and BC. Writing - review and editing, DM, CS, AC-R, SB, AH, FK, MZ, TKa, TKo, AB, SM, UJ, and FT. Visualization, NT. Supervision, DM, SM, UJ, and FT. All authors contributed to the article and approved the submitted version.

## Funding

This work has been funded by the “Brigitte & Dr. Konstanze Wegener” foundation.

## Conflict of Interest

TK holds stock of Roche AG. AH has received a research grant from the “Walter Schulz” foundation and advisory board, speech honoraria and travel expenses from Roche and Pfizer. AB has received advisory board and honoraria from AstraZeneca, Clovis, Roche and Tesaro. Research support, advisory board, honoraria, and travel expenses from AstraZeneca, Clovis, Medac, MSD, Novartis, PharmaMar, Roche, Sensor Kinesis, Tesaro, Teva have been received by SM and from AstraZeneca, Medac, PharmaMar, Roche, Tesaro by FT.

The remaining authors declare that the research was conducted in the absence of any commercial or financial relationships that could be construed as a potential conflict of interest.

## Publisher’s Note

All claims expressed in this article are solely those of the authors and do not necessarily represent those of their affiliated organizations, or those of the publisher, the editors and the reviewers. Any product that may be evaluated in this article, or claim that may be made by its manufacturer, is not guaranteed or endorsed by the publisher.

## Abbreviations

ACTBL2, Actin beta-like 2; BrdU, 5-bromo-2-deoxyuridine; BRCA1, breast cancer gene 1; cDNA, complementary deoxyribonucleic acid; CI, confidence interval; Cc, correlation coefficient; CPT1, carnitine palmitoyltransferase 1; Ct, cycle threshold; DAB, 3,3’diaminobenzidine; DNA, deoxyribonucleic acid; EOC, epithelial ovarian cancer; FAO, fatty acid oxidation; FBS, fetal bovine serum; FFPE, formalin-fixed and paraffin-embedded; GAPDH, glycerinaldehyd-3-phosphat-dehydrogenase; ICC, immunocytochemistry; IHC, immunohistochemistry; IRS, immunoreactive score; FIGO, International Federation of Gynecology and Obstetrics; LMU, Ludwig-Maximilians-University; mRNA, messenger ribonucleic acid; MTT, 3-(4,5-dimethylthiazol-2-yl)-2,5-diphenyltetrazolium bromide; MCR, Munich Cancer Registry; NFAT5, Nuclear factor of activated T-cells 5; OD, optical density; OS, overall survival; PBS, phosphate-buffered saline; qPCR, quantitative polymerase chain reaction; RIPA, radioimmunoprecipitation assay; ROC, receiver operating characteristic; RNA, ribonucleic acid ; RT, room temperature; siRNA, small interfering ribonucleic acid; TBS, tris-buffered saline; VSMCs, vascular smooth muscle cells; WHO, World Health Organization.
