# Supplementary material for: Actin Beta-Like 2 as a New Mediator of Proliferation and Migration in Epithelial Ovarian Cancer
Source: Front Oncol. 2021 Sep 23;11:713026. doi: 10.3389/fonc.2021.713026 (PMC8495414; doi:10.3389/fonc.2021.713026)
Supplement: Supplementary file 1 [file DataSheet_1.docx]

Supplementary Material


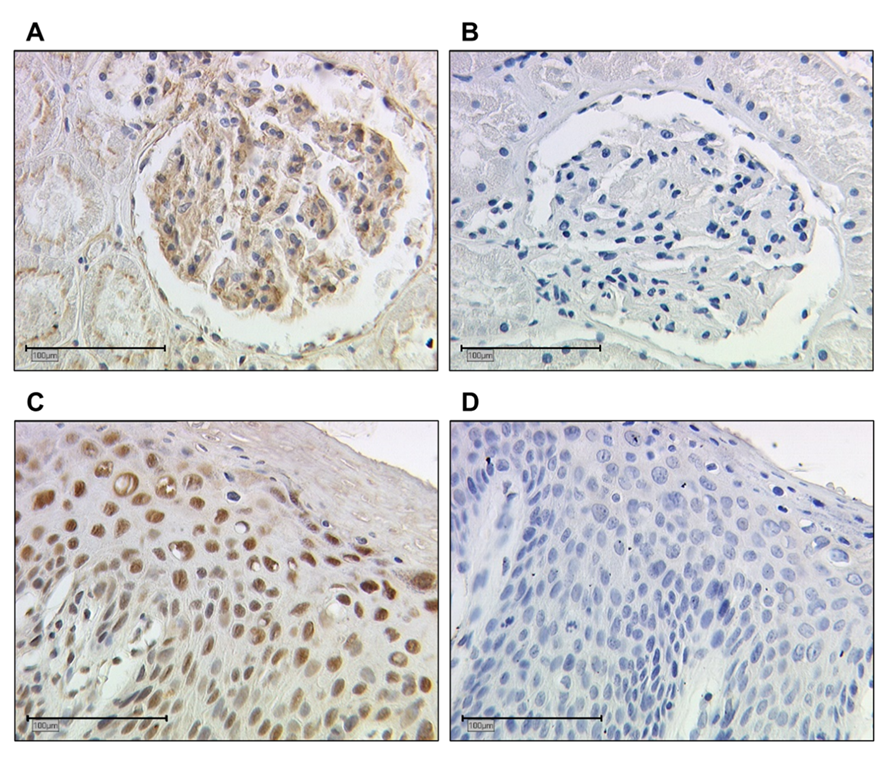


**Figure S1| Positive and negative system controls for ACTBL2 and NFAT5 in immunohistochemistry. (A, B)** Kidney tissue serving as positive (A) and negative (B) system control for ACTBL2 immunostaining. **(C, D)** Vulva tissue serving as positive (C) and negative (D) system control for NFAT5 immunostaining. (25x magnification; scale bar=100µm).


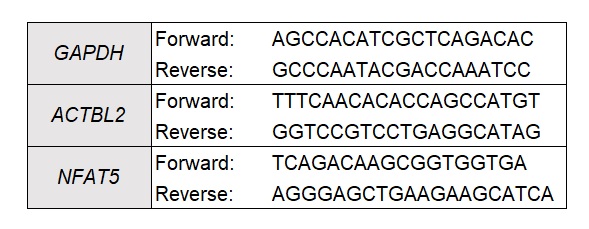


**Table S1| Primer sequences for qPCR.** Sequences of all qPCR primers (Roche, Basel, Switzerland) used in this study for determination of mRNA levels of *GAPDH, ACTBL2* and *NFAT5* in EOC cell lines.


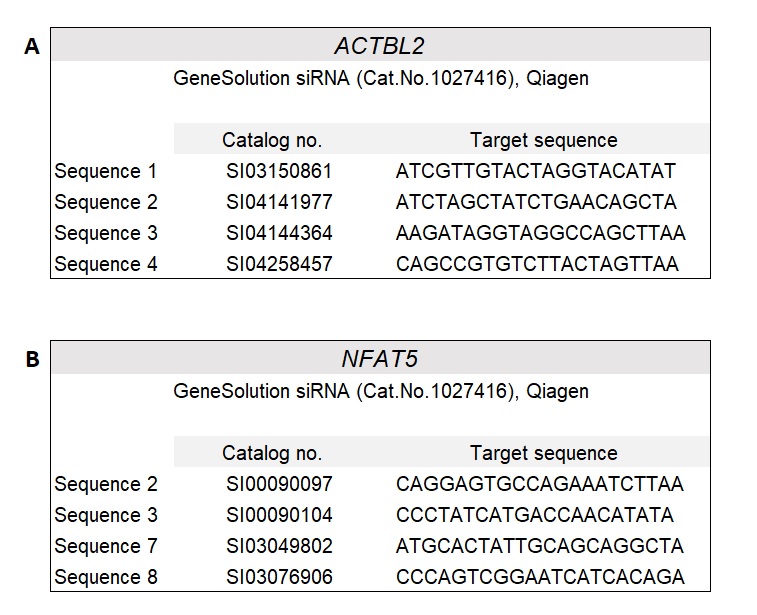


**Table S2| Catalog numbers and sequences of siRNA used in this study for (A) *ACTBL2* and (B) *NFAT5* knockdown, respectively.**


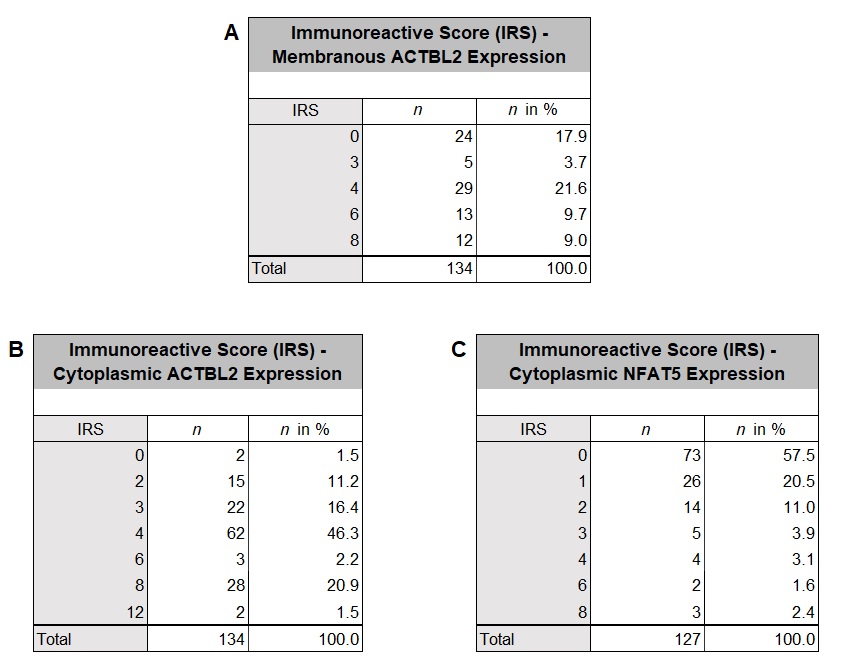


**Table S3| Descriptive statistics of Immunoreactive Scores (IRS) evaluated after immunohistochemical staining of ACTBL2 and NFAT5. (A)** Distribution of IRS regarding membranous ACTBL2 expression. **(B)** Distribution of IRS concerning cytoplasmic ACTBL2 expression. **(C)** Distribution of IRS regarding cytoplasmic expression of NFAT5.


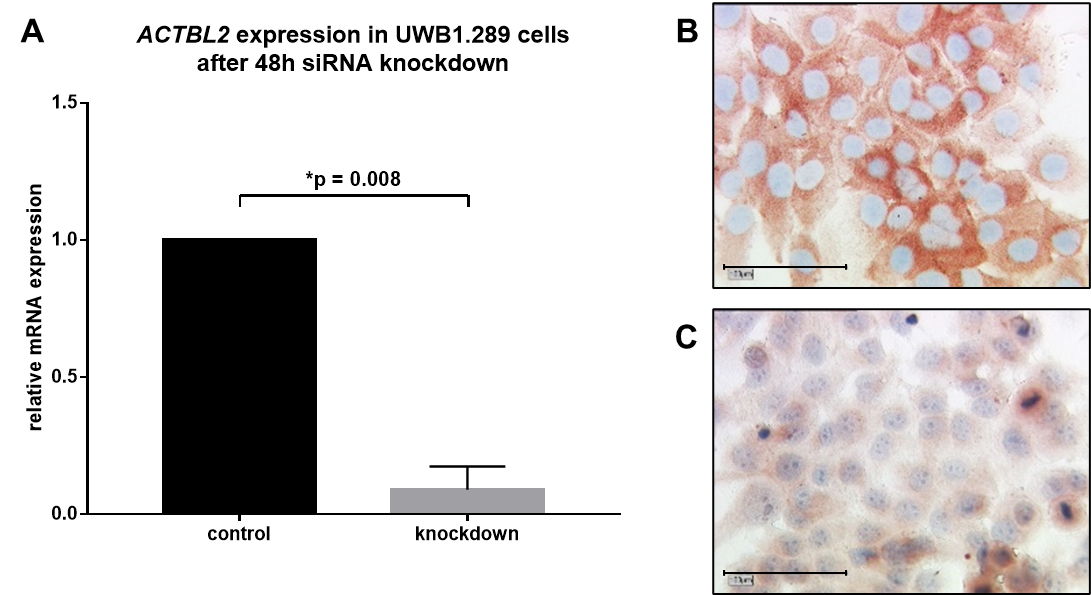


**Figure S2|** **Successful downregulation of *ACTBL2* by siRNA knockdown in UWB1.289 cells.**

**(A)** qPCR results of siRNA (sequence 3) knockdown of *ACTBL2* in UWB1.289 cells, proving a significant decrease (91%) in mRNA expression after 48h (*p*=0.008). **(B, C)** Immunocytochemistry of ACTBL2 in UWB1.289 cells after siRNA (sequence 3) knockdown of *ACTBL2*, showing a reduced protein expression after 72h of gene silencing (C) compared to the untreated control (B). (25x magnification; scale bar=100µm).


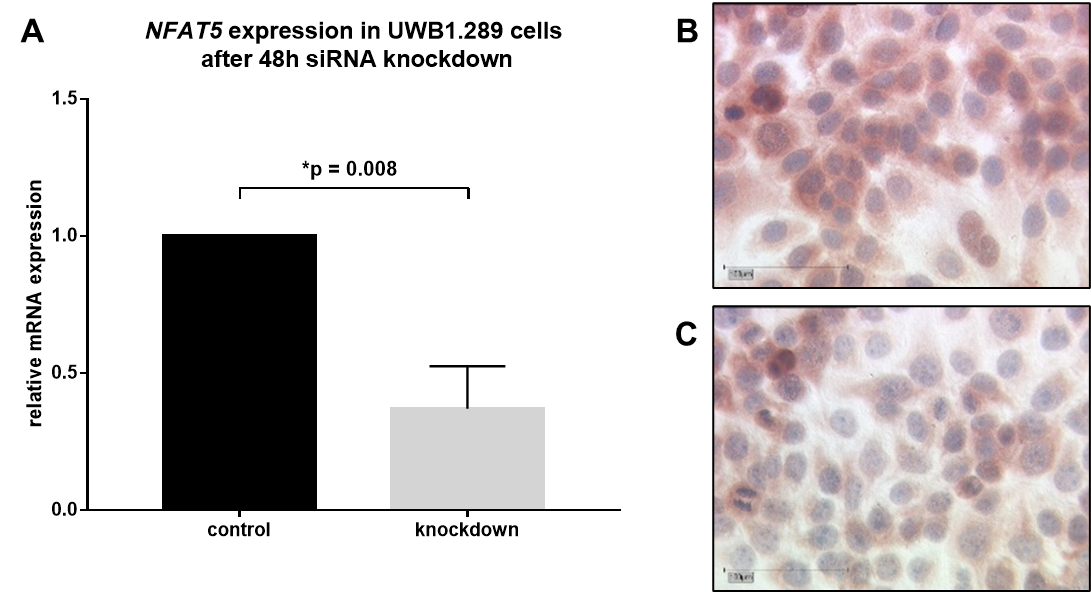


**Figure S3|** **Successful downregulation of *NFAT5* by siRNA knockdown in UWB1.289 cells.**

**(A)** qPCR results of siRNA (sequence 7) knockdown of *NFAT5* in UWB1.289 cells, proving a significant decrease (63%) in mRNA expression after 48h (*p*=0.008). **(B, C)** Immunocytochemistry of NFAT5 in UWB1.289 cells after siRNA (sequence 7) knockdown of *NFAT5*, showing a reduced protein expression after 72h of gene silencing (C) compared to the untreated control (B). (25x magnification; scale bar=100µm).


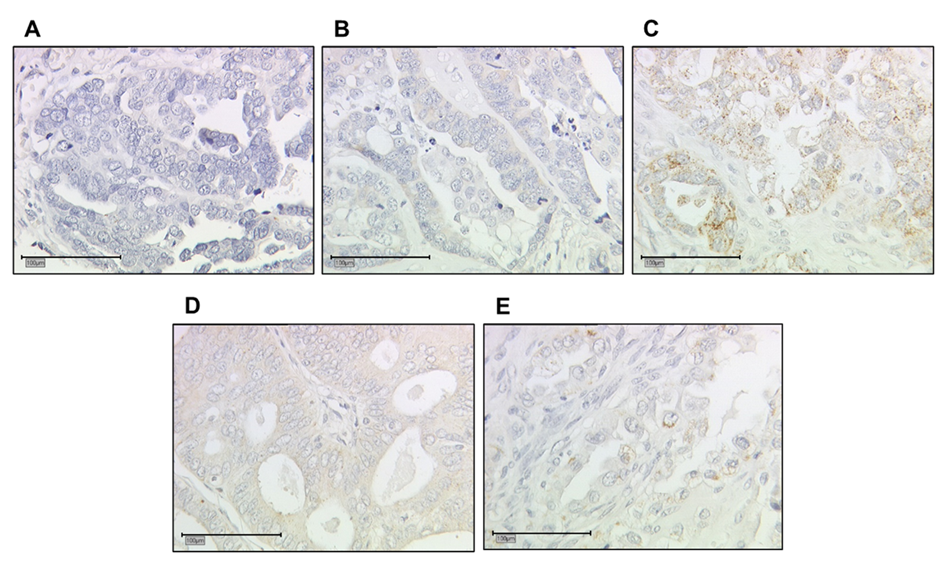


**Figure S4| Cytoplasmic NFAT5 expression in epithelial ovarian cancer.** Detection of NFAT5 by immunohistochemistry. Exemplary photographs (25x magnification; scale bar=100µm) showing cytoplasmic NFAT5 expression in different histological subtypes of ovarian cancer while comparing negative **(A)** to positive **(B-E)** NFAT5 expression: (A) endometrioid carcinoma, cytoplasmic IRS=0; (B) serous carcinoma, cytoplasmic IRS=1; (C) clear cell carcinoma, cytoplasmic IRS=8; (D) mucinous carcinoma, cytoplasmic IRS=8; (E) endometrioid carcinoma, cytoplasmic IRS=1.

|  | **Cytoplasmic NFAT5 expression** | |
| --- | --- | --- |
| **Variables** | **p** | **Correlation coefficient** |
|  |  |  |
| Histology | 0.212 | 0.111 |
| FIGO | 0.016* | -0.218 |
| pT | 0.002* | -0.268 |
| pN | 0.345 | -0.108 |
| Grading |  |  |
| *serous – low grading* | 0.015* | 0.216 |
| *serous – high grading* | <0.001** | -0.322 |
| *clear cell, endometrioid and mucinous – G1 to G3* | 0.240 | 0.107 |
|  |  |  |

**Table S4| Correlation analysis of cytoplasmic NFAT5 expression and clinicopathological data.** Spearman’s correlation analysis of cytoplasmic NFAT5 expression and clinicopathological characteristics, showing a negative correlation between high cytoplasmic IRS and FIGO status (*p*=0.016, *Cc*= -0.218). Moreover, cytoplasmic NFAT5 expression correlated negatively with high grading (*p*<0.001, *Cc*= -0.322) and positively with low grading (*p*=0.015, *Cc*=0.216) in serous carcinoma. Significant correlations are indicated with asterisks (*: *p*<0.05; **: *p*<0.001).

(*p*=two-tailed significance, *Cc*=correlation coefficient).


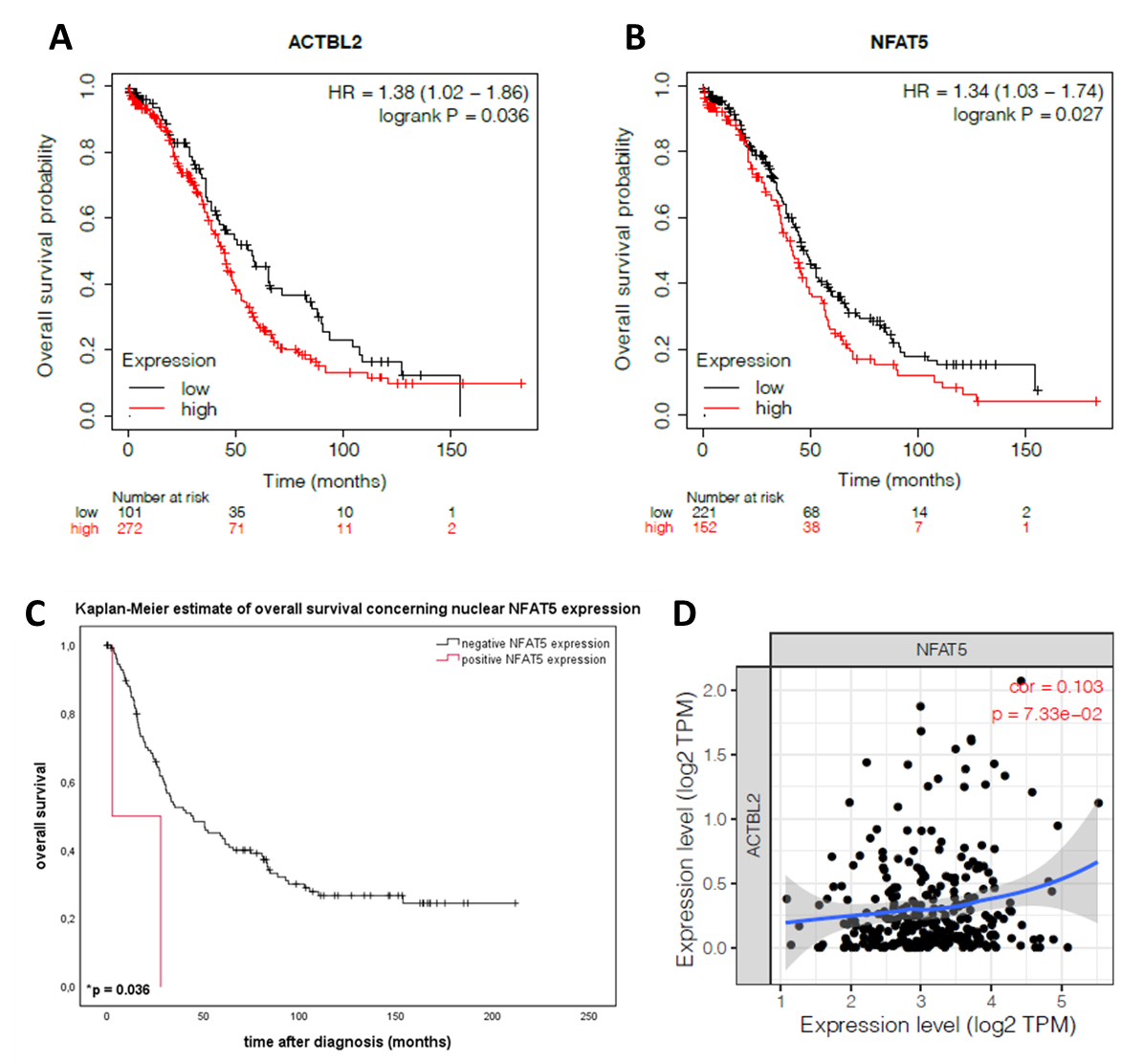


**Figure S5| Additional analyses of *ACTBL2* and *NFAT5* expression as well as their correlation using public databases. (A)** Kaplan-Meier plot concerning the gene expression of *ACTBL2* and overall survival using the KM plotter database (n_high_=272 vs. n_low_=101 patients; *p*=0.036).

**(B)** Kaplan-Meier plot concerning the gene expression of *NFAT5* and overall survival using the KM plotter database (n_high_=152 vs. n_low_=221 patients; *p*=0.027). **(C)** Kaplan-Meier plot concerning nuclear NFAT5 protein expression after immunohistochemical staining executed on tissue from our patient cohort (n_positive_=2 vs. n_negative_=129; *p*=0.036). **(D)** Scatter plot showing a positive correlation trend between gene expression of *ACTBL2* and *NFAT5* using the TIMER database (Spearman’s rho analysis; *p*=0.073, *Cc*=0.103).
